# Mechanism of Shenghua decoction in the treatment of primary dysmenorrhea based on network pharmacology and molecular docking analysis technology

**DOI:** 10.1097/MD.0000000000045879

**Published:** 2025-11-14

**Authors:** Yuwei Gu, Siyi Chen, Songhao Wu, Liang Xiong, Liuying Li, Zhonghui Pu

**Affiliations:** a School of Laboratory Medicine, Chengdu Medical College, Chengdu, China; b School of Pharmacy, Chengdu University of Traditional Chinese Medicine, Chengdu, China; c Department of Traditional Chinese Medicine, Zigong first People Hospital, Zigong, Sichuan, China.

**Keywords:** blood components, molecular docking, network pharmacology, potential mechanism, primary dysmenorrhea, Shenghua decoction

## Abstract

Primary dysmenorrhea (PD) is one of the common gynaecological disorders, which significantly affects the physical and mental health of women of reproductive age. Shenghua Decoction (SHD) is a classic formula for treating gynaecological disorders. However, the exact mechanism remains unclear, and there is limited research available on this topic. This study investigated the mechanism of SHD in the treatment of PD using network pharmacology and molecular docking techniques. The active ingredients of SHD were obtained from the *Radix Angelicae sinensis*, *Rhizoma chuanxiong*, *Semen persicae*, *Radix Glycyrrhizae* and *Rhizoma zingiberis preparata* of blood components for previous literature. Target prediction was performed by SwissTargetPrediction database, and PD-related disease targets were searched in Drugbank, Gene Cards, TTD and DisGeNET database. The protein–protein interaction (PPI) network was constructed using the STRING database and analyzed by Cytoscape 3.10.0 softwore. Additionally, the target genes were subjected to biological enrichment analysis in the Metascape database, including gene ontology (GO) and Kyoto encyclopedia of genes and genomes (KEGG) enrichment analysis. With the assistance of AutoDockVina and PyMOL software, a validation of molecular docking results and a visualization of the results were performed. This study identified 26 retained active ingredients of SHD, 484 drug targets, 81 of which were related to PD. GO enrichment analysis mainly involved 1173 biological processes such as response to hormone and positive regulation of cell migration. KEGG enrichment analysis mainly involved 141 pathways such as Steroid hormone biosynthesis, TNF and PI3K-Akt signaling pathway. Molecular docking showed that the core active ingredients of SHD, including liquiritigenin, ferulic acid and senkyunolide I, had strong binding abilities with core targets such as PTGS2 and AKT1. SHD can play a role in the treatment of PD through multi-component, multi-target hormone regulation, anti-inflammatory and analgesic pharmacological effects, which provide reference for subsequent research.

## 1. Introduction

Primary dysmenorrhea (PD) is defined as pain occurring with menses in the absence of pelvic pathology.^[1]^ Before and after menstruation or during menstruation, patients with dysmenorrhea usually have abdominal cramping, backache, bulge, accompanied by headache, nausea and even syncope in severe cases. PD seriously affects the life quality and work of patients. Epidemiological studies show that the incidence of dysmenorrhea in China is 77.1%, among which PD accounts for 90%. PD is a complex pathological process, involving the neuro-endocrine-immune network. Generally, it is believed that PD is related to endocrine disorders. The abnormal secretion of prostaglandins (PGs), particularly PGF_2α_ and PGE_2_, which promote uterine hypercontractility and ischemia.^[2]^ Current management of PD relies on nonsteroidal anti-inflammatory drugs (NSAIDs), such as aspirin, ibuprofen and naproxen sodium, which are the inhibitors of cyclooxygenase, are most frequently used for dysmenorrhea. However, NSAIDs have many side effects, particularly gastrointestinal disturbances and nephrotoxicity. In China, traditional Chinese medicines (TCMs), have been used for a long time to prevent and treat PD. Accumulating experimental and clinical evidence supports the efficacy of multi-component, multi-target TCMs with favorable safety profiles in preventing and managing PD^.[3,4]^ Some TCMs such as *Corydalis* yanhusuo demonstrate therapeutic effects through COX-2-mediated prostaglandin synthesis suppression, PGF_2α_/PGE_2_ ratio modulation, voltage-gated calcium channel blockade and μ-opioid receptor activation, and NF-κB pathway inhibition to reduce pro-inflammatory cytokine release, collectively improving the endometrial microenvironment.^[5,6]^

**Figure 1.**
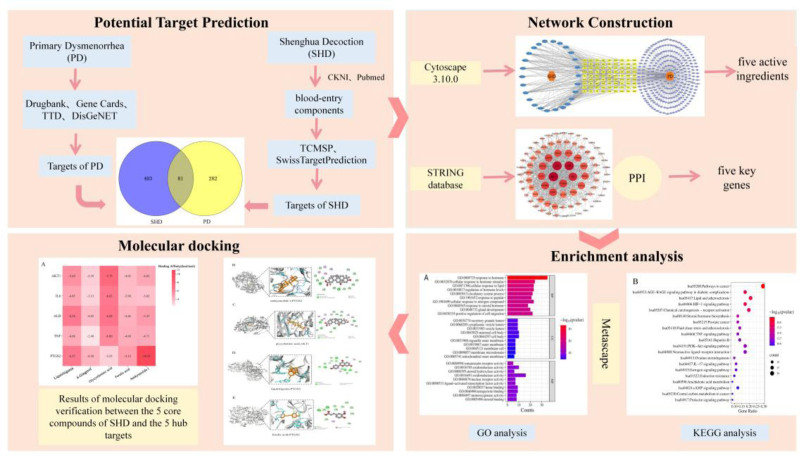
Specific process network pharmacology analysis.

Shenghua Decoction (SHD) is a well-known classic herbal formula documented in traditional Chinese medicine (TCM) that has been widely used in treating postpartum and gynecological diseases for several years. It was recorded by a famous TCM physician Qingzhu Fu in “Fu Qingzhu NvKe,” comprising 5 commonly used natural herbs: *Radix Angelicae sinensis*, *Rhizoma chuanxiong*, *Semen persicae*, *Radix Glycyrrhizae* and *Rhizoma zingiberis preparata*.^[7]^ Previous studies demonstrated that SHD can promote blood circulation, improve uterine contraction, alleviate abdominal pain, expel lochia, and have a positive effect on uterine recovery and female health-related quality of life during the postpartum period.^[8]^ However, there has been no study published, to date, reporting the anti-primary dysmenorrhea of SHD, and its active ingredients, target and molecular mechanism of action are still unclear. Serum pharmacochemistry of TCMs shows that the pharmacological effect of oral drugs must depend on the effective substances entering the blood circulation.^[9]^ It is more objective and precise to interpret the mechanism of action by the composition of blood. Hence, this study focused on the blood-entering components, investigated the efficacy and mechanism of SHD in treating PD by network pharmacology and molecular docking, which provided a reference for subsequent research. The specific process analysis is shown in Figure 1.

## 2. Materials and methods

### 2.1. Network pharmacology methods

#### 2.1.1. Screening of blood-entry components and potential targets of SHD

The terms “Shenghua decoction” and “blood-entry components” were used as a search term in the CNKI, VIP database, China Patent Network, PubMed, Web of Science and other related database from 2010 to 2024. The molecular 2D structure diagrams in sdf format were obtained from the PubChem database^[10]^ (https://pubchem.ncbi.nlm.nih.gov/). The compounds were then imported into the SwissTargetPrediction database^[11]^ (https://swisstargetprediction.ch) was used for target prediction, with duplicate targets and targets with a probability of “0” being removed.

#### 2.1.2. Prediction of target genes for PD

To identify potential targets for PD, we searched for information in 4 online databases: the DrugBank^[12]^ (https://go.drugbank.com/), DisGeNET^[13]^ (https://www.disgenet.org/), Genecards^[14]^ (https://www.genecards.org/) and TTD^[15]^ (https://db.idrblab.net/ttd/). These databases contain information about human genes and genetic disorders, as well as potential therapeutic targets for diseases. We then integrated all the targets, removed duplicate data, obtaining PD-related potential valuable targets.

#### 2.1.3. The common targets overlapped between active compounds and PD-related targets

By identifying the common targets shared by PD-related targets and the predicted SHD targets, a visual representation was shown in a Venn diagram using the Venny 2.1.0 platform (https://bioinfogp.cnb.csic.es/tools/venny/)

#### 2.1.4. Construction of protein–protein interaction (PPI) network and Screenning the intersection target for PD

The genes that were known to be both drug targets and disease targets were imported into the STRING database^[16]^ (STRING: http://string-db.org), which is a tool for finding interacting genes and proteins. The database was set to the species “Homo sapiens” (human), and the confidence score for PPI was set to 0.4. Cytoscape software,^[17]^ version 3.10.0, was used to create a network of potential key targets. The CytoHubba plugin was then used to filter and select the core targets in the network. Core targets were identified based on 3 essential topological parameters: degree centrality (DC), closeness centrality (CC), and betweenness centrality (BC).

#### 2.1.5. Gene ontology (GO) and KEGG pathway enrichment analysis

The intersection targets were input into the Metascape database (https://metascape.org/gp/index.html) and the selected identifer was set as “Homosapiens.” The biological process (BP), cell component, molecular function (MF) and Kyoto encyclopedia of genes and genomes (KEGG) pathway were obtained and analyzed. The top 20 pathways were selected based on the ascending order of *p*-values and uploaded to a bioinformatics platform^[18]^ (http://www.bioinformatics.com.cn/) for visualization.

### 2.2. Organ localization analysis of the core target

The core targets were imported into BioGPS database (http://biogps.org/), and the species was selected as human to obtain the gene expression dataset of the relevant genes in different tissues and organs, and the organ expression heatmap of the core targets of SHD blood components for PD treatment was generated by using the website of ChiPlot (https://www.chiplot.online/). The darker the color in the heat map, the more genes were expressed and distributed in the tissue.

### 2.3. Molecular docking

To validate the predictions derived from network pharmacology and substantiate the interactions between key targets and compounds, molecular docking analysis was further conducted. In this study, we employed 3D structures of the key target proteins were downloaded through the PubChem database (https://pubchem.ncbi.nlm.nih.gov/), and the proteins were subjected to the removal of water of crystallisation and hydrogenation using the AutoDockTools 1.5.6 software; developed and maintained by The Scripps Research Institute, La Jolla, and then the molecular docking and visualization of the docking results were performed using the AutoDock vina 1.1.2 and the Pymol2.1; developed and maintained by Schrödinger LLC, New York, Discovery studio (DS) V2019 (Dassault Systemes Biovia, San Diego) software for molecular docking and visualization of docking results. The results were evaluated in terms of the level of binding free energy as an evaluation of the degree of binding to the compound; generaly the lower the energy of the compound molecule when it is conformationally stable for binding to the receptor, the stronger the binding that occurs.

## 3. Results

### 3.1. Network pharmacology study

#### 3.1.1. Blood-penetrating ingredients of SHD and obtaining the SHD component-PD disease potential targets

**Figure 2.**
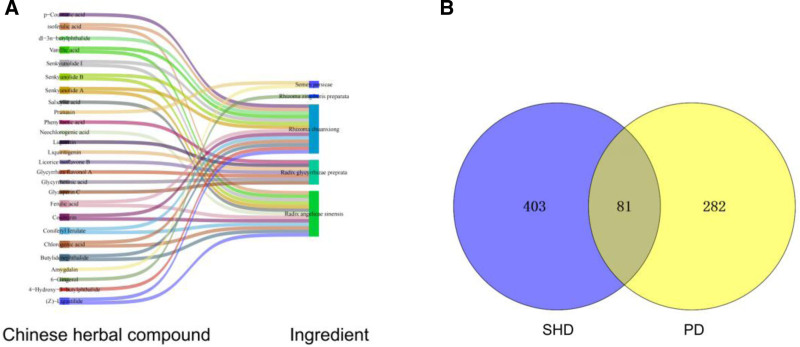
Screening of SHD active compounds. (A) Sankey diagram of associations between Chinese herbal compounds and ingredients in SHD. (B) Venn diagram of 81 common targets between active compound targets of SHD and PD disease targets. PD = primary dysmenorrhea, SHD = Shenghua decoction.

According to the literature^[19,20]^ a total of 26 blood-penetrating components of SHD were obtained, among which 13 were in Radix Angelicae sinensis, 14 were in Rhizoma chuanxiong, 2 were in Semen persicae, 1 were in Radix Glycyrrhizae and 7 were in Rhizoma zingiberis preparata Radix Angelicae (Table 1). A visualization in the form of a Sankey diagram was generated to illustrate the associations between Chinese herbal compounds and ingredients in SHD using bioinformatics platform (Fig. 2A). These blood-penetrating ingredients were then imported into the Swiss Target Prediction databases for target prediction, resulting in the retention of 484 drug targets. A search was conducted in the Drubank, GeneCards, TTD, Disgenet databases, resulting in the retention of 363 PD targets. The PD targets were uploaded to the bioinformatics platform to generate a Venn diagram (Fig. 2B). A total of 81 intersecting targets were obtained by comparing the PD targets with the SHD targets.

**Table 1:** Basic information of constituents migrating to blood of Shenghua decoction.

MOL ID	Chemical composition	Molecular	Source
MOL000431	Coumarin	C_9_H_6_O_6_	Radix angelicae sinensis, rhizoma chuanxiong
MOL003871	Chlorogenic acid	C_16_H_18_O_9_	Radix angelicae sinensis, rhizoma chuanxiong
MOL000360	Ferulic acid	C_10_H_10_O_4_	Radix angelicae sinensis, rhizoma chuanxiong
MOL000114	Vanillic acid	C_8_H_8_O_4_	Radix angelicae sinensis, rhizoma chuanxiong
MOL005928	Isoferulic acid	C_10_H_10_O_4_	Radix angelicae sinensis, rhizoma chuanxiong
MOL008288	Coniferyl ferulate	C_20_H_20_O_6_	Radix angelicae sinensis, rhizoma chuanxiong
MOL002111	Butylidenephthalide	C_12_H_12_O_2_	Radix angelicae sinensis, rhizoma chuanxiong
MOL002122	(Z)-Ligustilide	C_12_H_14_O_2_	Radix angelicae sinensis, rhizoma chuanxiong
MOL002143	Senkyunolide I	C_12_H_16_O_4_	Radix angelicae sinensis, rhizoma chuanxiong
MOL002208	Senkyunolide A	C_12_H_16_O_2_	Radix angelicae sinensis, rhizoma chuanxiong
MOL011784	Senkyunolide B	C_12_H_12_O_3_	Radix angelicae sinensis, rhizoma chuanxiong
MOL003066	Neochlorogenic acid	C_16_H_18_O_9_	Radix angelicae sinensis
MOL001801	Salicylic acid	C_7_H_6_O_3_	Radix angelicae sinensis
MOL000771	p-Coumaric acid	C_9_H_8_O_3_	Rhizoma chuanxiong
MOL002181	4-Hydroxy-3-butylphthalide	C_12_H_14_O_3_	Rhizoma chuanxiong
MOL002189	dl-3n-butylphthalide	C_12_H_14_O_2_	Rhizoma chuanxiong
MOL001320	Amygdalin	C_20_H_27_NO_11_	Semen persicae
MOL006221	Prunasin	C_14_H_17_NO_6_	Semen persicae
MOL002467	6-Gingerol	C_17_H_26_O_4_	Rhizoma zingiberis preparata
MOL009294	Phenyllactic acid	C_9_H_10_O_3_	Radix glycyrrhizae preprata
MOL004903	Liquiritin	C_21_H_22_O_9_	Radix glycyrrhizae preprata
MOL001792	Liquiritigenin	C_15_H_12_O_4_	Radix glycyrrhizae preprata
MOL004811	Glyasperin C	C_21_H_24_O_5_	Radix glycyrrhizae preprata
MOL005008	Glycyrrhiza flavonol A	C_20_H_18_O_6_	Radix glycyrrhizae preprata
MOL004884	Licorice isoflavone B	C_20_H_16_O_6_	Radix glycyrrhizae preprata
MOL004804	Glycyrrhetinic acid	C_30_H_46_O_4_	Radix glycyrrhizae preprata

#### 3.1.2. Construction of SHD component and PD disease intersection targets network

**Figure 3.**
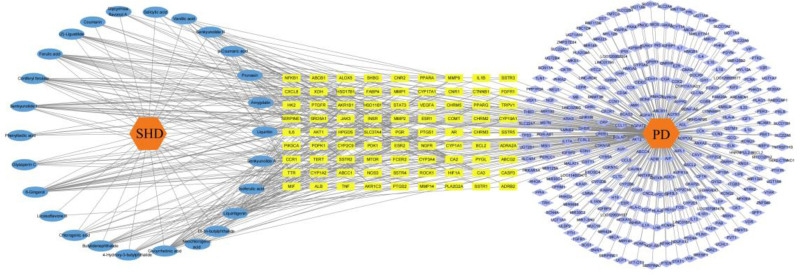
Constituents migrating to blood-targets network. The blue circles respresents the constituent migrating to blood; the yellow hexagons respresents the intersection target of blood component and PD; the pink diamond represents the PD gene. PD = primary dysmenorrhea.

26 active ingredients were imported into Cytoscape 3.10.0 software for topological analysis, along with 81 intersecting targets. The network consisted of 391 nodes and 661 edges (Fig. 3). By sorting them based on their degree values, liquiritigenin (degree = 25), 6-gingerol (degree = 23), glycyrrhetinic acid (degree = 21), ferulic acid (degree = 20) and senkyunolide I (degree = 19) were selected. It is speculated that these 5 active ingredients may be the core effective components of SHD in treating PD.

#### 3.1.3. Construction of PPI interaction regulatory network of SHD and PD intersection targets

**Figure 4.**
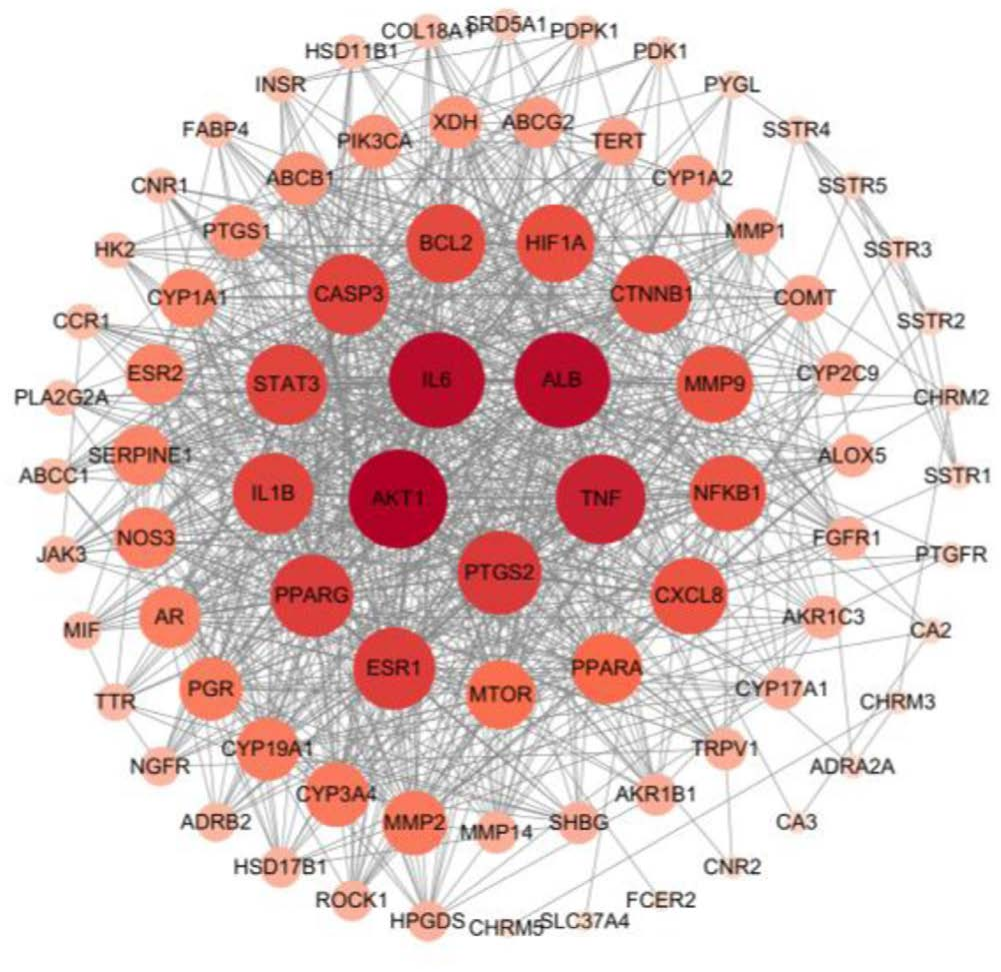
PPI network of target protein. PPI = protein–protein interaction.

The intersection targets were imported into the STRING databases to construct the PPI network for the treatment of PD with SHD. A total of 81 (representing target proteins) nodes and 805 edges (indicating interactive relationships) were obtained, with an average node degree of 20. Nodes represent the targets, the edges represent the interactions between proteins, and the different colors represent different interactions. Visual analysis was performed using the Cytoscape3.10.0 software (Fig. 4). The top 5 genes were selected based on their topological parameters degree of connectivity (DC), CC, BC and identifed as key genes. These key genes include AKT1, IL6, ALB, TNF and PTGS2, as shown in Table 2 It is speculated that these genes may play a crucial regulatory role in the treatment of PD with SHD.

**Table 2:** Key targets of SHD in the treatment of PD.

Target	DC	CC	BC
AKT1	53	0.72727	0.077833
IL6	51	0.72727	0.078598
ALB	51	0.70796	0.072738
TNF	47	0.68376	0.033389
PTGS2	43	0.66116	0.030729

DC = degree centrality, CC = closeness centrality, BC = betweenness centrality, PD = Primary dysmenorrhea, SHD = Shenghua decoction.

#### 3.1.4. Go and KEGG enrichment analysis

**Figure 5.**
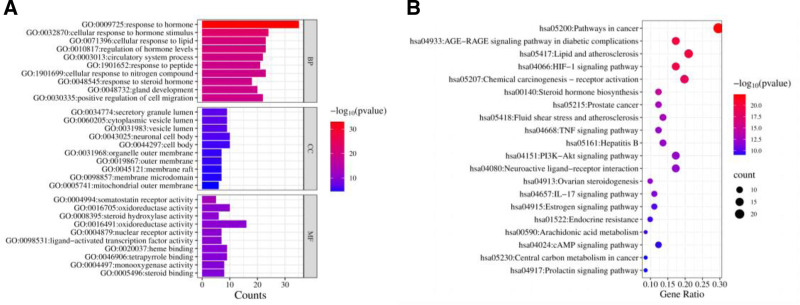
The results of functional analysis of common targets between SHD and PD. (A) the gene ontology (GO) functional analysis, highlighting the enriched biological processes, cellular components, and molecular functions associated with the common targets. (B) The Kyoto Encyclopedia of Genes and Genomes (KEGG) pathway enrichment analysis, revealing the pathways that are significantly enriched among the common targets. GO = gene ontology,

A total of 1333 GO entries were enriched in the Metascape database, 1173 of which were BP, 114 were MF, and 46 were CC, accounting for 88%, 9% and 3%, respectively. The most signifcant BP involved response to hormone (GO:0009725), cellular response to hormone stimulus (GO:0032870), cellular response to lipid (GO:0071396) and regulation of hormone levels (GO:0010817). The most signifcant CC involved secretory granule lumen (GO:0034774), cytoplasmic vesicle lumen (GO:0060205), vesicle lumen (GO:0031983) and neuronal cell body (GO:0043025). The most signifcant MF terms included somatostatin receptor activity (GO:0004994), oxidoreductase activity (GO:0016705), steroid hydroxylase activity (GO:0008395) and oxidoreductase activity (GO:0016491). The top 10 significantly enriched terms were screened by the BP, cellular component (CC) and MF categories (Fig. 5A).

**Figure 6.**
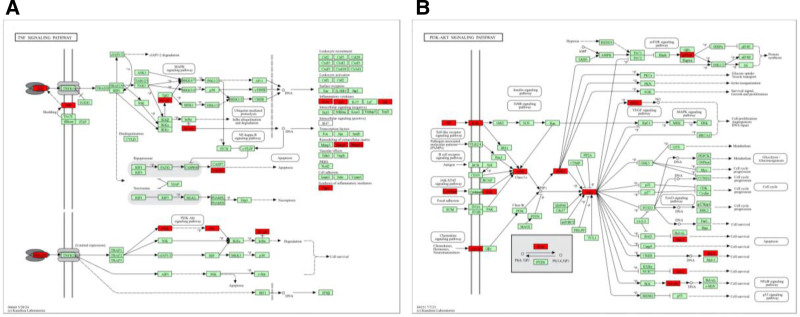
Potential target spots of SHD on regulating TNF and PI3K-Akt. Arrows indicate activation effects, and T-arrows indicate inhibition effects. The spots in red represent targets regulated by SHD. SHD = Shenghua decoction.

The KEGG enrichment analysis mainly involved 141 signaling pathways (Fig. 5B), the top 20 signaling pathways were selected and imported into the online program microscopic letter (http://www.bioinformatics.com.cn). It was found that 9 signaling pathways may be related to the treatment of PD with SHD, including the teroid hormone biosynthesis (hsa00140), TNF signaling pathway (hsa04668), PI3K-Akt signaling pathway (hsa04151), Ovarian steroid production (hsa04913), IL-17 signaling pathway (hsa04657), Estrogen signaling pathway (hsa04915), Arachidonic acid metabolism (hsa00590), cAMP signaling pathway (hsa04024) and Prolactin signaling pathway (hsa04917). Furthermore, within the TNF signaling pathway and PI3K-Akt signaling pathway, SHD demonstrates modulation on a predominant set of target proteins and exerts influence over several sub-pathways, as depicted in Figure 6 Collectively, these findings suggest that SHD may mitigate PD via a mechanism characterized by its “multi-compounds, multi-targets, and multi-pathways” approach.

### 3.2. The target-organ analysis

**Figure 7.**
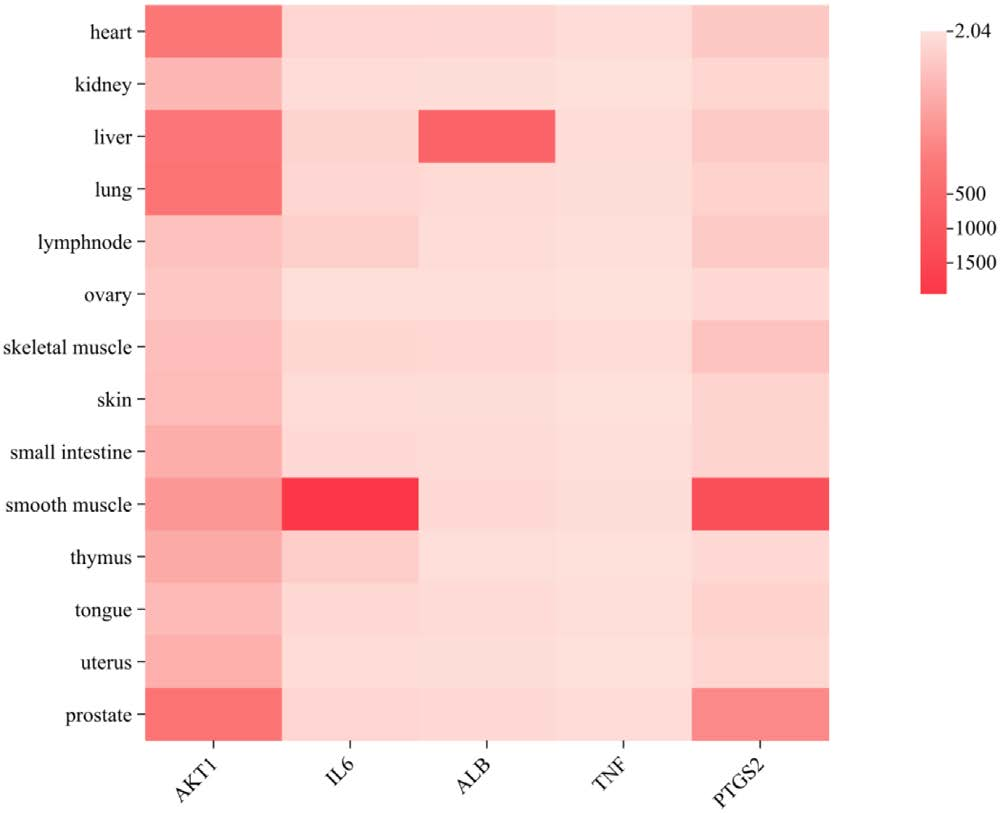
Distribution of core targets in organs and tissues.

Effort was invested in evaluating mRNA levels for all 5 core targets, utilizing data sourced from the BioGPS database to analyze the metabolic process of SHD anti-PD in vivo. Chinese medicine believes that PD is closely related to the function of the uterus, liver, kidneys and spleen, while Western medicine believes that PD is related to the prostate gland. The core target points of SHD for treating PD are distributed in tissues and organs such as heart, liver, smooth muscle, uterus and prostate (see Fig. 7), indicating that SHD not only conforms to the Chinese medicine theory of sparing the liver and regulating qi, activating blood circulation and removing blood stasis to relieve pain, but also conforms to the practice of Western medicine, which has significant advantages for treating PD.

### 3.3. Molecular docking

**Figure 8.**
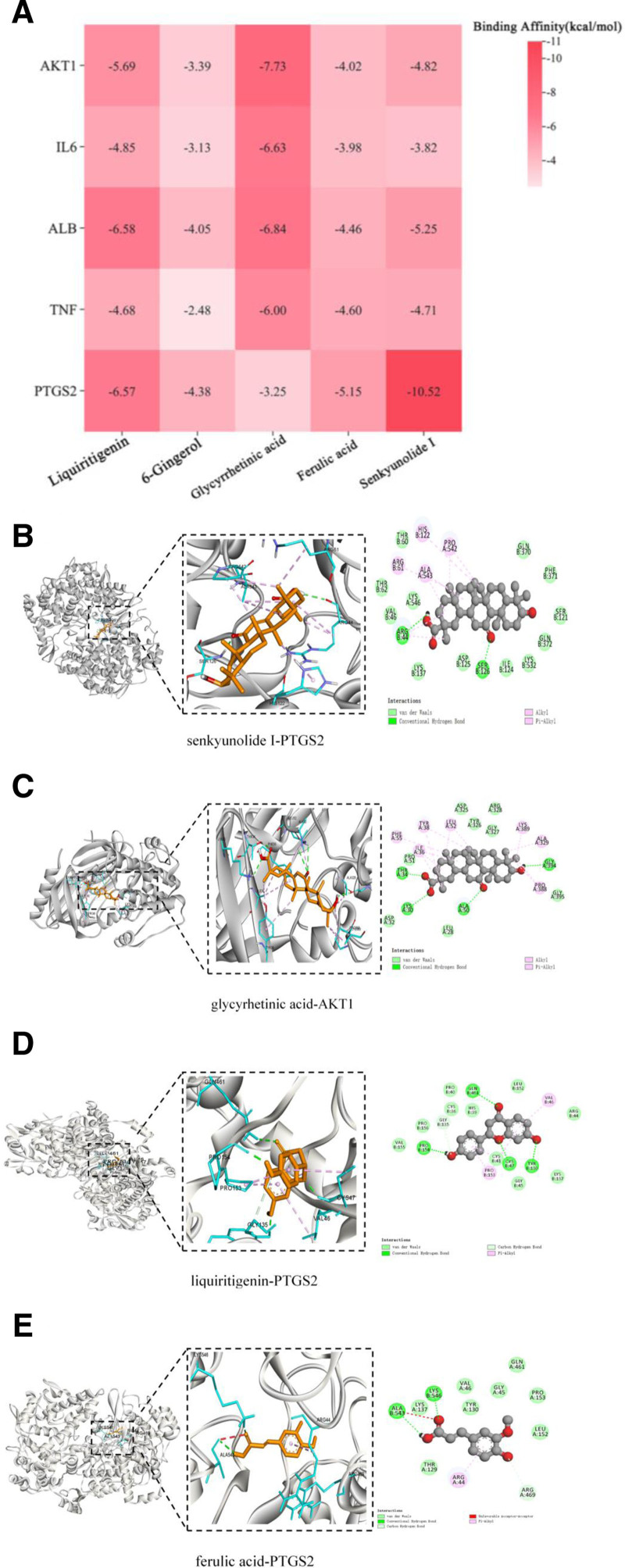
Results of molecular docking verification between the 5 core compounds of SHD and the 5 hub targets. (A) Heatmap of affinities. (B) Molecular docking of senkyunolide I and PTGS2. (C) Molecular docking of glycyrrhetinic and AKT1. (D) Molecular docking of liquiritigenin and PTGS2. (E) Molecular docking of ferulic acid and PTGS2. SHD = Shenghua decoction.

Five core ingredients from SHD were linked with AKT1 (4EJN), IL6 (1A1U), ALB (1N5U), TNF (5UUI), and PTGS2 (5F19) targets. The docking of each active component and protein produces 25 docking results (Fig. 8A). According to the principle of a lower binding energy corresponding to an increased possibility of interaction between protein and molecule and to a stable binding conformation, the conformation with the lowest binding energy of ligand and receptor is selected as the docking conformation.^[21]^ The results showed the binding energies of liquiritigenin, ferulic acid, senkyunolide I and PTGS2 target were all less than −5 kcal mol^−1^, indicating that the energetic drug molecules could bind to the target protein well. The conformation with the lowest binding energy of ligand and receptor was selected as the docking conformation. In the findings presented in Figure 8B to E, senkyunolide I established hydrogen bonds with PTGS2 at residues ARG-44, SER-126. Ferulic acid formed hydrogen bonds with PTGS2, specifically at ALA-543 and LYS-546, while liquiritigenin engaged with PTGS2 at PRO-154, GLN-461, CYS-47 and TYR-130. Hydrogen bonding of glycyrrhetinic acid was evident with AKT1 at LYS-30, THR-34, GLY-394 and ALA-50. These interactions emphasize the intricate molecular relationships between the active compounds and respective protein targets. These computer-simulation results of molecular docking confirmed the network pharmacology analysis on how SHD components influence PD and laid the foundation for further study on its pharmacological mechanisms.

### 3.4. Ethical approval

As this analysis utilized only publicly available, preexisting data from public database searches and involved no human participants, animal experiments, or clinical trials, ethical approval was not required.

## 4. Discussion

In this study, an integrated strategy combining network pharmacology, blood-entry components of SHD and molecular docking was used. It could elucidate the possible effective active ingredients of SHD in PD quickly and clearly. Meanwhile, network pharmacology said 26 active ingredients of SHD blood-entry components, as well as 363 drug targets, 81 of which are related to PD. The core active ingredients are liquiritigenin, 6-gingerol, glycyrrhetinic acid, ferulic acid, and senkyunolide I, while the core targets are AKT1, IL6, ALB, TNF and PTGS2. Molecular docking results showed that the core active ingredients and core targets can bind well, indicating reliable docking results. Among them, senkyunolide I, glycyrrhetinic acid, liquiritigenin and ferulic acid have the low hydrogen bonding affinity with the core targets, suggesting that they may be the core substances underlying the treatment of PD with SHD. This 4 components can exert multiple pharmacological effects such as anti-inflammatory and analgesic effects by regulating the expression of inflammatory factors, modulating hormone levels, and inhibiting uterine smooth muscle contraction or uterine inflammatory response.^[22,23]^

The key target AKT1, also known as protein kinase B, belongs to the family of serine/threonine kinases that regulate processes such as metabolism, proliferation, growth, and angiogenesis.^[24]^ Studies have shown that inhibiting the activity of AKT1 can effectively improve the pain symptoms of endometriosis and the inflammatory response to relieve the pain symptoms of PD. II-6 has a contractile effect on the uterus, which triggers an inflammatory response, leading to ischemia and hypoxia in the local tissue of the uterine epithelium, causing PD.^[25]^ ALB is synthesized by hepatic parenchymal cells and plays an important role in maintaining blood colloid osmotic pressure, scavenging free radicals, and other biological processes closely related to systemic nutrient status and inflammatory response. It also has an important modulator function in PD.^[26]^ TNF is an important inflammatory factor and has immunomodulatory effects that can act on tissue cells, stimulate cell growth, and promote extracellular matrix (ECM) proliferation. PTGS2 is the body’s rate-limiting enzyme that catalyzes the synthesis of arachidonic acid and releases PGF2α. Activation of the PGF2α produced, triggered by inflammatory factors, leads to a spasmodic contraction of the uterine smooth muscle, resulting in PD.^[27]^

Based on the KEGG signaling pathways and GO prediction of BP pathway, MF pathway and CC pathway by Metascape, the GO results indicated that SHD could affect hormones, cell migration and hypoxia by treating PD. The KEGG pathway is mainly enriched in signaling pathways such as steroid hormone biosynthesis, TNF, PI3K-Akt, ovarian steroidogenesis, IL-17, and estrogen. Steroid hormones play a crucial role in stabilizing the homeostasis of the microenvironment around the oocyte and relieving pain in PD.^[28]^ The TNF signaling pathway promotes the expression of genes such as pro-inflammatory factors, chemokines, and TNF-α. The IL-17 signaling pathway inhibits the production of inflammatory factors and chemokines and relieves inflammation.^[29]^ The key targets of IL6, ALB, TNF, and PTGS2 are enriched in the TNF signaling pathway and IL-17 signaling pathway. The PI3K-Akt signaling pathway is widely distributed in various tissues and organs of the body. By inhibiting the activation of the PI3K/AKT signaling pathway, it can downregulate the levels of downstream effectors, COX-2 and VEGF, thereby inhibiting endometrial vascular proliferation and glandular growth and improving the symptoms of PD.^[30]^ The estrogen signaling pathway can promote uterine smooth muscle and vasodilation and reduce harmful messages by regulating the expression of ET-1 and NO.^[31]^

The overproduction of prostaglandins causes the narrowing of the blood vessels supplying the uterus, hypercontractility of the uterus (cramps) which leads to ischemia, hypoxia of the uterus, and increased sensitivity of the nerve endings.^[32]^ Molecular docking showed that the strongest binding targets of senkyunolide I, liquiritigenin, and ferulic acid were PTGS2. The expression level of PTGS2 was induced to be up-regulated by cytokines and other cytokines, which catalyzed the production of arachidonic acid to produce a variety of metabolites such as prostaglandins.^[33]^ Whether SHD treats PD by acting on PTGS2 targets and the specific mechanism of action need to be further studied.

According to traditional Chinese medicine theory, dysmenorrhea is often caused by poor circulation of qi and blood and deficiency of nourishment to the uterus. SHD has the effects of nourishing blood, promoting blood circulation, removing blood stasis and generating new blood, as well as warming the meridians and relieving pain. Through the combination of multiple TCMs, it acts on multiple systems and targets of the human body, regulating the balance of qi and blood in women as a whole. In contrast, NSAIDs mainly alleviate uterine smooth muscle spasms and inflammatory responses by inhibiting the activity of cyclooxygenase (COX) and reducing the synthesis of prostaglandins, thereby achieving the purpose of pain relief. Its mechanism of action is relatively simple, only intervening in a certain link where pain occurs, and it cannot comprehensively regulate the female body. Numerous clinical studies have shown that SHD had good efficacy in the treatment of PD. In a study involving 120 patients with PD, the patients were randomly divided into 2 groups. The control group was treated with prostaglandin synthesis inhibitors, while the treatment group was treated with modified SHD on the basis of the control group. The results showed that the total effective rate of the treatment group reached 90.0%, which was significantly higher than 73.3% of the control group.^[34]^

The study may provide clues for further study of SHD treatment on PD. However, there are limitations in the present study, the mechanism pathway is still in the prediction stage, which needs to be further verified by experiments. To comprehensively understand the mechanism of SHD on PD, we will carry out corresponding experiments in the following work.

## 5. Conclusion

In this study, network pharmacology, blood-entry components of SHD and molecular docking were used to predict and validate the therapeutic effect of SHD. Network pharmacological analysis, molecular docking and literature predicted that SHD is associated with prostaglandins and some biomarkers related to inflammation, which were crucial for SHD treating PD.

In summary, this research focuses on the multi-ingredient and polybiological function property nature of TCM. It is the first time to comprehensively analyze of the association between the SHD active ingredient and PD target, which can provide more data support and theory support for the anti-PD mechanism of SHD.

## Acknowledgments

We thank all authors for their contributions and support.

## Author contributions

**Conceptualization:** Yuwei Gu, Zhonghui Pu.

**Data curation:** Siyi Chen, Songhao Wu.

**Formal analysis:** Liang Xiong, Liuying Li.

**Funding acquisition:** Zhonghui Pu.

**Investigation:** Yuwei Gu, Siyi Chen, Songhao Wu.

**Methodology:** Liang Xiong, Liuying Li, Zhonghui Pu.

**Project administration:** Yuwei Gu.

**Resources:** Zhonghui Pu.

**Software:** Yuwei Gu, Siyi Chen, Songhao Wu.

**Supervision:** Liang Xiong, Liuying Li, Zhonghui Pu.

**Validation:** Yuwei Gu, Siyi Chen.

**Visualization:** Yuwei Gu, Siyi Chen, Songhao Wu, Liang Xiong, Liuying Li, Zhonghui Pu.

**Writing – original draft:** Yuwei Gu.

**Writing – review & editing:** Yuwei Gu, Zhonghui Pu.
